# Low Medium pH Value Enhances Anthocyanin Accumulation in *Malus* Crabapple Leaves

**DOI:** 10.1371/journal.pone.0097904

**Published:** 2014-06-10

**Authors:** Yanchen Zhang, Jie Zhang, Tingting Song, Jinyan Li, Ji Tian, Kaina Jin, Yuncong Yao

**Affiliations:** 1 Plant Science and Technology College, Beijing University of Agriculture, Beijing, China; 2 Key Laboratory of New Technology in Agricultural Application of Beijing, Beijing University of Agriculture, Beijing, China; Institute of Botany, Chinese Academy of Sciences, China

## Abstract

Anthocyanin is a critical factor involved in coloration of plant tissues, but the mechanism how medium pH values affect anthocyanin accumulation in woody plants is unknown. We analyzed anthocyanin composition and the expression of elements encoding anthocyanin and flavonols biosynthesis underlying different medium pH values by using three different leave color type cultivars. HPLC analysis demonstrated that high medium pH values treatment induced a dramatic decrease in the concentration of cyaniding in crabapple leaves. Conversely, the high medium pH values induced up-regulation of the content of flavones and flavonols, suggesting that low pH treatment-induced anthocyanin accumulation. Quantitative real time PCR experiment showed the expression level of *anthocyanidin synthase* (*McANS*) and *uridine diphosphate glucose flavonoid 3-O-glucosyltransferase* (*McUFGT*) was up-regulated by low pH values treatment, and high medium pH value treatment up-regulate the transcription level of *flavonol synthase* (*McFLS*). Meanwhile, several MYB TFs have been suggested in the regulation of pH responses. These results strongly indicate that the low pH treatment-induced anthocyanin accumulation is mediated by the variation of mRNA transcription of the anthocyanin biosynthetic genes.

## Introduction

Anthocyanins are plant secondary metabolites that are responsible for the characteristic red, blue, and purple color of plant tissues. Anthocyanins also play an important role in plant reproduction, by attracting pollinators and seed dispersers, and also in protection from stress including photo-oxidative stress [1].

In most species, the coloration of plant tissues results from the accumulation of anthocyanins pigments in the vacuoles of (sub) epidermal cells, and anthocyanins change their color depending on the pH of the vacuole in which anthocyanins localize; their color is bluer in weakly acidic or neutral pH, and redder in acidic pH [2], [3].

In morning glory (*Ipomoea tricolor*) petals, the vacuolar pH is relatively low when the flower bud opens, resulting in a red color, but upon further maturation, the vacuolar pH increases and the petals acquire a strong blue color [4]. This color change and the increase of vacuolar pH require a putative Na^+^/H^+^ exchanger encoded by the *PURPLE* gene [5]. Most likely, *PURPLE* transports sodium ions into and protons out of the vacuole, resulting in a less acidic vacuole and a bluer color.

*Petunia hybrida* flowers normally have a lower pH than Ipomoea flowers, and the color of wild-type flowers stays on the reddish (low pH) side of the color spectrum. *PH4* encodes a MYB domain protein that is expressed in the petal epidermis, mutation of *PH4* results in a bluer flower color, increased pH of petal extracts, and, in certain genetic backgrounds, the disappearance of anthocyanins and fading of the flower color [6]. Mutations in the genes *ANTHOCYANIN1* (*AN1*), *AN2*, and *AN11* cause, besides the loss of anthocyanin pigments, an increased pH of petal extracts [7]. But there were few reports about the effect of environmental pH values on plant tissues color.

*Malus* crabapples are one of the most important ornamental and economic germplasm resources, providing abundant plant landscape species and favorable research material to exploit the mechanism of color formation, which is due to the diverse colors of its leaves, flowers and fruits as a result of different anthocyanin materials [8]. Leave color is an important ornamental trait of *Malus* crabapples, and the color of the leaves differs in different varieties. ‘Flame’, ‘Prairifire’ and ‘Royalty’ are three typical crabapple varieties and represent major leave color characteristics of crabapple. The leaves of the ‘Flame’ are green throughout the development, the leaves of ‘Prairifire’ turns green from red during development, while the leaves of ‘Royalty’ change from red to purple during development [9].

In this study, different medium pH treatment triggered the alteration of flavonoid contents was investigated in three *Malus* crabapple cultivars. Our findings have provided strong evidence that the low pH treatment-induced anthocyanin accumulation. Flavonoid/anthocyanin biosynthetic and regulation genes respond environmental pH values by controlling anthocyanins accumulation in *Malus* crabapple leaves.

## Results

### The effect of different pH to the development of crabapple seedlings

**Figure 1:**
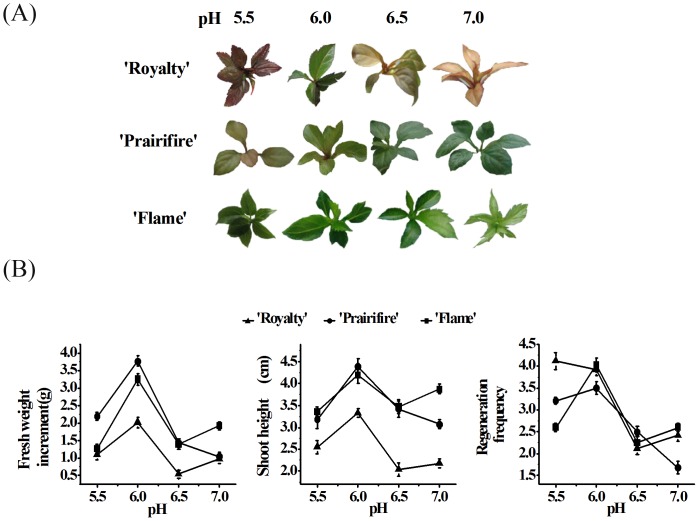
Different medium pH values can alter plant development during plant tissue culture of *Malus* crabapple. (A) Seedlings harvested from the different medium pH values treatment. (B) The fresh weight increment, shoot height and regeneration frequency of the crabapple seedlings were analyzed in different medium pH values.

In order to detect how medium pH values affect seedlings development of each cultivar, we made 4 different kinds of pH values to conduct adventitious buds regeneration experiment. Multiple coefficients were determined after 30 days cultured in different medium (Fig. 1 A).

Figure 1B showed that the fresh weight increment, shoot height and regeneration frequency of three crabapple cultivars ‘Flame’, ‘Royalty’, and ‘Prairifire’ were significantly higher than other pH treatments when pH was 6.0. Meanwhile, we found that the growth of seedlings were inhibited by 6.5 and 7.0 pH treatments, and the three coefficients of 6.5 and 7.0 pH values were obviously lower compared with 5.5 and 6.0 pH values. These results suggested that 6.0 was the most suitable pH values for the growth of crabapple seedlings, and the neutral and alkaline environment would inhibit the development of crabapple.

### Leaf coloration respond to environmental pH values variation in crabapple leaves

**Figure 2:**
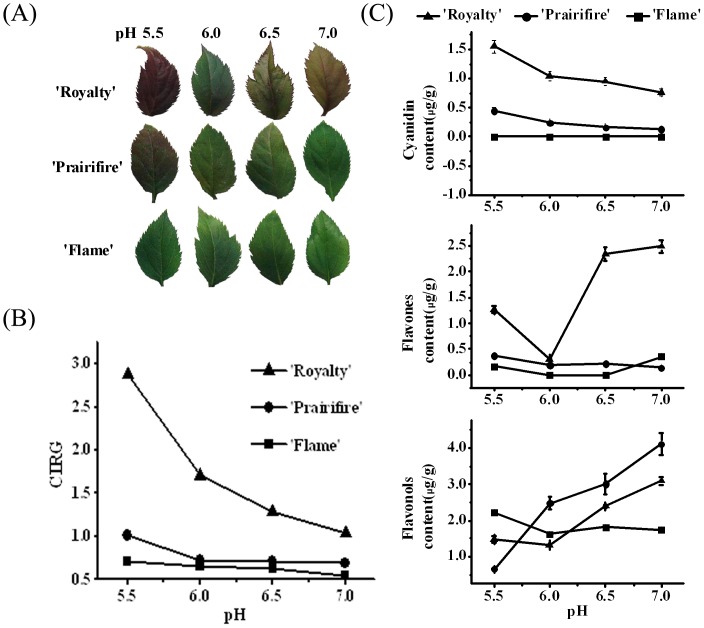
Analysis of color variation of crabapple leaves. (A) Phenotype of the leaves from three crabapple cultivars after different medium pH values treatment. (B) Color measurement by Minolta chromameter, shown as CIRG values. A shift from zero towards greater numbers indicates a color change from green towards red. (C) HPLC analysis for anthocyanins, flavonols and flavones in the leaves of three *Malus* crabapple cultivars after different medium pH values treatment. Error bars on each symbol are mean ± SE of three replicate reactions.

To explore the relationship between environmental pH values and crabapple anthocyanins variation, we conducted the leaf color measurement and the HPLC analysis of crabapple leaves in different pH values medium. As described above, crabapple cultivars ‘Flame’, ‘Royalty’, and ‘Prairifire’ are related plants but with a rather different leaf color. As shown in Fig. 2 A, increased medium pH values induced the leaf color to shift from red to green in ‘Royalty’, and ‘Prairifire’, but there's no obvious variation in ‘Flame’.

The variations of leaf color during leaf development were quantified using CIRG value. The results showed that the CIRG values of ‘Royalty’ and ‘Prairifire’ were gradually decreased with the increased of pH values, and the CIRG values of ‘Flame’ maintained a relative low level compared with another two cultivars. The CIRG values also suggest the leaf color of ‘Royalty’ were shifted to yellow in pH 6.5 and 7.0 (Fig. 2 B).

The phenolic compounds are responsible for color. Anthocyanins are one type of flavonoids, other flavonoids include flavonols, flavones. Cyanindin is one of anthocyanins and provide various red colours in plant. Flavonols and flavones are largely responsible for the production of yellow color. HPLC analysis showed that the major anthocyanins in crabapple leaves was cyanindin, and high medium pH (7.0) reduced the total cyanindin content to less than half of that in the low medium pH (5.5) in ‘Royalty’ and ‘Paraitfire’ leaves. On the contrary, increased pH value (from 6.0 to 7.0) up-regulate the content of flavones which produce yellow colors in ‘Royalty’ leaves, and the content of flavonols in ‘Royalty’ and ‘Paraitfire’ were also enhanced by increased pH treatment.

The main flavonoids components in green varieties ‘Flame’ were flavonols and there was no significantly variation with different medium pH treatments (Fig. 2 C). Taken together, these results suggest that changed medium pH value triggered pigmentation variations are mediated by the variation of flavonoids contents and component.

### The effect of different pH to the expression level of anthocyanin/flavonoid biosynthetic genes

To examine the molecular mechanisms how high medium pH values treatment induce anthocyanins accumulation in crabapple leaves An attempt was made to assess whether the changed medium pH values induced flavonoid biosynthetic genes transcription alterations in leaves.

**Figure 3:**
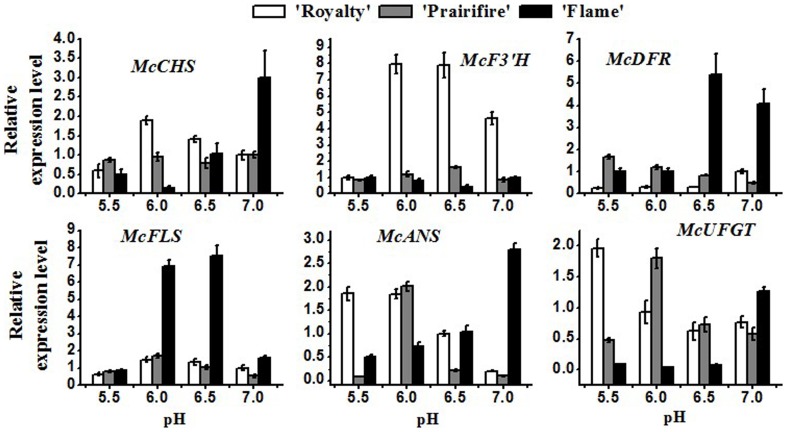
Expression profile of *Malus* crabapples anthocyanin biosynthetic genes during different medium pH values treatment. Real-time PCR was used to analyze *McCHS*, *McF3′H*, *McDFR*, *McFLS*, *McANS*, *McUFGT* expression patterns in leaves of *Malus* crabapple cultivars ‘Royalty’, ‘Prairifire’ and ‘Flame’. *Malus* crabapple 18S ribosomal RNA gene was used as the reference gene. Error bars on each symbol are mean ± SE of three replicate reactions.

Transcripts levels of anthocyanin biosynthetic structural genes *McANS* and *McUFGT*, are response for the last reaction in anthocyanin biosynthetic pathway, were significantly higher in ‘Royalty’ and ‘Prairifire’ in acidic medium than ‘Flame’, with the highest transcript levels at pH 5.5. The expression levels of these genes were consistent with the variation of anthocyanins content and the degree of pigmentation observed during pH values variation (Fig. 3).

On the contrary, the expression of *McDFR* (dihydroflavonol 4-reductase), *McF3′H* (flavonoid 3′-monooxygenase) and *McFLS* which control biosynthetic direction to either anthocyanin or flavonol pathway, encoding anthocyanin or flavonols biosynthesis was increased 5- to 30-fold in ‘Flame’ than in another two cultivars in alkalinity conditions (Fig. 3), and the content of flavonols were examined in ‘Flame’ had the same accumulation tendency. This result strongly suggests that higher pH induced flavonols accumulation in ‘Flame’ are mediated by the variation of *McDFR* and *McFLS* expression.

The relative analysis showed the expression level of *McDFR* has close relationship with cyanidin, flavonols and flavones in crabapple cultivar ‘Prairifire’. Meanwhile, the expression of *McANS* had an approximate variation profile with the content of these three flavonoid components in ‘Royalty’. On the other side, the content of flavonols in ‘Flame’ was consisted with the expression pattern of *McF3′H*, and the content of flavones was consisted with the expression of *McCHS* (chalcone synthase), *McANS* and *McUFGT*. To sum up, the different flavonoid material in three crabapple cultivars were synthesized by different anthocyanin biosynthetic genes (Table 1).

**Table 1:** Correlation of *Malus* leaves flavonoids content and anthocyanin biosynthetic gene expression under different acidity.

Flavonoid	Varieties	*McCHS*	*McF3′H*	*McFLS*	*McDFR*	*McANS*	*McUFGT*
Cyanidin	Royalty	−0.5	−0.68	−0.61	−0.43	0.79	0.95
	Prairifire	−0.27	−0.43	0.02	0.96**	−0.06	−0.15
	Flame	-	-	-	-	-	-
Flavonols	Royalty	−0.21	0.09	−0.05	0.48	−0.99**	−0.58
	Prairifire	0.44	0.23	−0.17	−0.99**	−0.02	0.06
	Flame	−0.21	0.33	−0.63	−0.27	−0.38	−0.24
Flavones	Royalty	−0.41	−0.06	−0.22	0.65	−0.86	−0.39
	Prairifire	−0.56	−0.23	−0.10	0.89*	−0.28	−0.35
	Flame	0.83	0.72	−0.54	0.34	−0.79	−0.89

Statistical comparison was performed by t test: *P>0.05, **P>.01.

### The effect of different pH to the expression level of anthocyanin/flavonoid regulation genes

**Figure 4:**
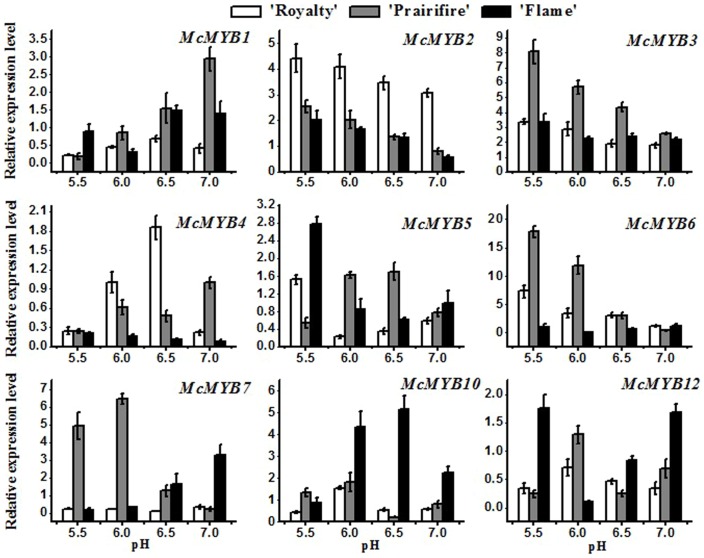
Expression profile of *Malus* crabapples anthocyanin regulation genes during different medium pH values treatment. Real-time PCR was used to analyze *McMYBs* expression patterns in leaves of *Malus* crabapple cultivars ‘Royalty’, ‘Prairifire’ and ‘Flame’. *Malus* crabapple 18S ribosomal RNA gene was used as the reference gene. Error bars on each symbol are mean ± SE of three replicate reactions.

MYB transcription factors have been shown to play an important role in the transcriptional regulation of anthocyanins in apple, so we detected the expression profile of this transcription factors by different pH treatment in three cultivars (Fig. 4), and the relative analysis were calculated in Table 2. The results showed that cyaniding were positive regulated by *McMYB6* in ‘Royalty’ and ‘Prairifire’ (0.99 and 0.95, respectively), and *McMYB2* and *McMYB3* also involved in the regulation process of cyaniding biosynthesis in ‘Royalty’ (0.87 and 0.90, respectively). For flavonols, there are different main regulation MYB transcription factors among three crabapple cultivars, the expression trend of *McMYB5* was consistent with the accumulation of flavonols in ‘Flame’ (0.91). On the other hand, flavonols concentration were positive regulated by *McMYB1* and *McMYB4* (0.94 and 0.91, respectively) and negative regulated by *McMYB3* and *McMYB6* (−0.99 and −0.95, respectively) in ‘Prairifire’. In addition, the *McMYB2* and *McMYB3* transcript levels negative correlated with flavonols concentration in ‘Royalty’ (−0.95 and −0.90, respectively). Meanwhile, the content of flavones was also negative regulated by *McMYB2* and *McMYB3* in ‘Royalty’ (−0.80 and −0.80, respectively). Levels of *McMYB3* and *McMYB4* positive and negative associated with flavones biosynthesis in ‘Prairifire’, respectively (0.92 and −0.91). Interestingly, the expression profile of *McMYB6* showed a same spatial and temporal pattern with the accumulation of flavones in ‘Flame’ (0.82).

**Table 2:** Correlation of *Malus* leaves flavonoids content and anthocyanin regulation gene expression under different acidity.

Flavonoids	Varieties	*McMYB1*	*McMYB2*	*McMYB3*	*McMYB4*	*McMYB5*	*McMYB6*	*McMYB7*	*McMYB10*	*McMYB12*
Cyanidin	Royalty	−0.65	0.87	0.90	−0.28	0.83	0.99**	−0.22	−0.19	−0.17
	Prairifire	−0.86	0.92	0.96*	−0.81	−0.50	0.95	0.66	0.50	−0.29
	Flame	-	-	-	-	-	-	-	-	-
Flavonols	Royalty	0.36	−0.95*	−0.90	−0.04	−0.25	−0.74	0.39	−0.51	−0.56
	Prairifire	0.94	−0.97*	−0.99**	0.91	0.31	−0.95*	−0.71	−0.46	0.29
	Flame	0.17	0.90	0.96*	0.58	0.91	0.56	−0.04	−0.72	0.69
Flavones	Royalty	0.37	−0.80	−0.80	0.09	−0.02	−0.45	0.10	−0.77	−0.73
	Prairifire	−0.08	0.84	0.92	−0.91	−0.44	0.83	0.46	0.21	−0.55
	Flame	0.43	0.10	0.10	−0.37	0.27	0.82	0.66	−0.71	0.81

Statistical comparison was performed by t test: *P>0.05, **P>0.01.

## Discussion

pH values is one of important factors which affect anthocyanins accumulation. It has been previously proposed that the suitable pH for anthocyanins copigmentation is pH 3.5 [10], and the Na+/H+-antiporter in Japanese morning glory (*Ipomea nil*) and PH4 in Petunia can alter the anthocyanins accumulation [5], [6], [11], which activates vacuolar acidification. However, in comparison with vacuolar pH- induced anthocyanins accumulation, relatively little is known about the role of plant tissue culture pH variation in anthocyanins accumulation response.

In recent years, more and more attention has been paid to the breeding of color-leafed (especially red/purple leaf) plants for ornamental use [12]. Red-leaf color is one of the most important factors for ornamental landscape use, which can provide a perfect visible sense and take a great cultivation advantage due to its stress resistance. Due to leaves, flowers and fruits have diverse colors and polyphenolics compounds, *Malus* crabapple is becoming an excellent model plant to uncover the pigmentation mechanism [9]. In this paper, we focused on the mechanism of the medium pH values could affect the anthocynins accumulation between plant tissue culture.

Our results showed that cyanindin, flavonols and flavones have different patterns of accumulation in three cultivars of *Malus* crabapple during pH treatment. The medium pH values had no obviously effect on the cyanidin accumulation and the content of flavonols in alkalinity mediums were enhanced in ever-green cultivar ‘Flame’. Flavonols biosynthesis mainly depends on the expression of *McFLS* [13] (Fig. 3), Therefore, we speculated that the high expression level of flavonols biosynthetic genes in ‘Flame’ is the main reason for flavonols accumulation under high pH values condition in this cultivar (Fig. 2 C). These results consist with the previous analysis of flavonols accumulation [14], [15]. Meanwhile, the expression profile of the *McANS* and *McUFGT* gene was significantly different from all the other examined biosynthetic genes. Low pH treatment led to an increase in abundance of the *McANS and McUFGT* transcript, the pattern of expression is correlated with the pattern of cyanidin accumulation in leaves (Fig. 3). So we thought that acidic environmental condition can enhanced cyanidin biosynthesis through activate the expression of these two genes in colored-leaves cultivars [16]–[18] (Fig. 2 C). And our results indicated there was no cyanidin accumulation in leaves of ‘Flame’ (noncolored-leaves cultivars) with low pH treatments, due to multiple anthocyanin pathway steps were deficient in the leaves of non-colored cultivars [19]–[21].

Based on previous studies, it seems likely that variation in activity of the R2R3 MYB TFs is responsible for controlling the spatial and temporal patterning of anthocyanin production in most plant species [15], [21], [22]–[24]. In our work, the expression level of several MYB transcription factors involved in the regulation of flavonoids synthesis under different pH treatment conditions. In ‘Royalty’, *McMYB2* and *McMYB3* were likely to promote cyanidin and inhibit flavonol and flavones biosynthesis during pH values variation in crabapple leaves, and *McANS* and *McUFGT* maybe involved in this regulation process. Relative expression analysis in ‘Prairifire’ revealed the variation of *McMYB3* accompanied by a corresponding shift in the expression levels of *McDFR*, so we deduced that *McDFR* maybe a candidate downstream gene of *McMYB3* which plays an important role in the flavonoids biosynthesis. Flavonols is the main flavonoids component in ‘Flame’ leaves, qRT-PCR results showed that abundant expression of *McMYB5* can activate the expression of *McDFR* and *McFLS* and promote flavonol synthesis with pH variation. To sum up, different MYB TFs functions as regulators in different leaf color crabapple cultivars after pH induction.

In summary, our study showed low environmental pH values is able to promote anthocyanins accumulation in colored-leaves cultivars by activate the expression of late anthocyanin biosynthetic genes, and high pH values is able to increase the content of flavonols in non-colored -leaves cultivars through the abundant expression of *McFLS*. Furthermore, McMYB TFs plays an important role in modulating plant coloration in crabapple in different pH medium, mainly through transcriptional regulation of its downstream genes in the anthocyanin biosynthetic pathway. Undoubtedly the work described in this report will provide a convenient way to regulate plant color by altering environmental pH values.

## Materials and Methods

### Plant material

The plant materials used for the tissue culture included three crabapple varieties: (1) *Malus* ‘Flame’ (both young and mature leaves are green), (2) *Malus* ‘Prairifire’ (young leaves are orange to red and mature leaves are green) and (3) *Malus* ‘Royalty’ (both young and mature leaves are red to purple). These 5-year-old crabapple trees grafted on *Malus* ‘Balenghaitang’ were planted in Crabapple Germplasm Resources Nursery in Beijing University of Agriculture.

Tissue culture plantlet of *Malus* ‘Flame’, *Malus* ‘Prairifire’ and *Malus* ‘Royalty’ were preserved at tissue culture center of Beijing University of Agriculture. Cultural conditions: temperature was 23±2°C, humidity was 60%–70%, light intensity was 1800–2000 Lux with 16 h light a day.

### pH treatment

The adventitious buds which were approximate 1.5 cm long and collected from regeneration medium were harvested and transferred to MS medium (Murashige and Skoog) with 2.2 µM 6-benzyladenine (6-BA) and 0.5 µM a-Naphthaleneacetic acid (NAA) with various pH values (5.5, 6.0, 6.5, 7.0), The increase of fresh weight, shoot height, regeneration frequency were determined after 30 days.

### Measurement of flavonoid content and leaf color

Quantification of pigments was performed by high-performance liquid chromatography (HPLC). One hundred milligrams of fresh petals was homogenized with a mortar and pestle under liquid nitrogen. Then 1 ml of extraction solution containing methanol∶ water ∶ formic acid∶ trifluoroacetic acid (70 ∶ 27 ∶ 2 ∶ 1, v/v).,and the mixture was stored overnight at 4°C in the dark. The mixture was centrifuged at 4°C at 15,000 rpm for 15 min, and The supernatants were filtered through a 0.22 µm Millipore TM filter before used. Anthocyanins in the samples were analyzed using a HPLC1100-DAD system (Agilent Technologies, Waldbronn, Germany).Detection was performedat 520 nm for anthocyanin, 350 nm for flavonoid. A NUCLEODURH C18 column (250 mm, 64.66 mm) (Pretech Instruments, Sollentuna, Sweden) was used for separation at 25°C and eluted using a mobile phase consisting of solvent A, trifluoroacetic acid ∶ formic acid∶ water (0.1∶ 2 ∶ 97.9) and solvent B, trifluoroacetic acid ∶ formic acid∶ acetonitrile∶ water (0.1∶ 2∶ 35∶ 62.9) at a flow rate of 0.8 ml min-1. The elution program was followed the procedure described previously with some modifications [25]. Solvent B was 30% initially 84 and increased linearly in steps to 35% at 5 min, 40% at 10 min, 50% at 30 min, 55% at 50 min, 60% at 70 min, 30% at 80 min. HPLC analysis was performed as described previously [26].

The flower color variables of both crabapple and tobacco were measured immediately after picking. L*, a*, and b* values were measured randomly using a Konica Minolta CR-400 Chroma Meter (Minolta, Japan). The value ‘L*’ represents brightness and darkness, ‘a*’ represents greenness and redness as the value increases from negative to positive, and ‘b*’ represents blueness and yellowness. The value ‘C*’ represents saturation and was calculated according to the formula C* = (a*2+b*2) 1/2. Hue angle (h*) was calculated according to the following equation: H = arctan (b*/a*) (McGuire, 1992). CIRG (Color Index for Red Grape) was calculated according to the formula CIRG = (180−H)/(L*+C) [27].

### Quantitative real-time PCR analysis

Total RNA from flower tissues was extracted using an RNA Extract Kit (Aidlab, Beijing, China) according to the manufacturer's instructions. DNase I (TaKara, Japan) was added to remove genomic DNA, and the samples were then subjected to cDNA synthesis using the Access RT-PCR System (Promega, USA) according to the manufacturer's instructions. The expression levels of *McCHS*, *McDFR*, *McF3′H*, *McFLS*, *McANS*, and *McUFGT* were analyzed using quantitative real-time PCR (RT-qPCR) with SYBR Green qPCR Mix (Takara, Japan) and Bio-Rad CFX96 Real-Time PCR Systems (BIO-RAD, USA), according to the manufacturers' instructions. The primers in this paper were designed by NCBI Primer BLAST and listed in Table 3.

**Table 3:** Oligo Primers.

Accession number	ID	Sequence (5′-3′)	Product length	Purpose
DQ341382	18SR-F	GAGCCTGAGAAACGGCTACC	102 bp	qRT-PCR
	18SR-R	GTCACTACCTCCCCGTGTCA		qRT-PCR
FJ599763	McCHS-F	TGACCGTCGAAGTTCGC	182 bp	qRT-PCR
	McCHS-R	TTTGTCACACATGCGCTGGA		qRT-PCR
KF481684	McF3′H-F	CGTTGCTGTCGCTCACGGATGA	108 bp	qRT-PCR
	McF3′H-R	ATGACGTGTCAGTGCCAGCTGTG		qRT-PCR
KF495602	McFLS-F	ACGAGCAACCGGGAATCACAACTG	120 bp	qRT-PCR
	McFLS-R	CCCAGTTGGAGCTGGCCTCAGTA		qRT-PCR
FJ817487	McDFR-F	CCGAGTCCGAATCCGTTTGT	126 bp	qRT-PCR
	McDFR-R	CCTTCTTCTGATTCGTGGGGT		qRT-PCR
FJ817488	McANS-F	CACAGGGGCATGGTGAACAA	202 bp	qRT-PCR
	McANS-R	TTCACTTGGGGAGCAAAGCC		qRT-PCR
KF495603	McUFGT-F	CGGGTGCAAATTCGGACCAAGGGT	194 bp	qRT-PCR
	McUFGT-R	ACGACCCATCTCGCTCGTCCCTCG		qRT-PCR
KJ126856	McMYB1-F	GCTCACACCAACAAAGGAGC	139 bp	qRT-PCR
	McMYB1-R	GCAGCTCTTCCCACATCGAA		qRT-PCR
KJ126857	McMYB2-F	GCTGCGATAAGCAGGACACAA	249 bp	qRT-PCR
	McMYB2-R	ACCGGTTCCCTAGGAGTGC		qRT-PCR
KJ126858	McMYB3-F	ACATTAAGACTCATGGGGAAGGC	207 bp	qRT-PCR
	McMYB3-R	GCAGCCTTCCAGCAATCAAC		qRT-PCR
JX013493	McMYB4-F	GGACCAGCAGCAGGAAACTA	161 bp	qRT-PCR
	McMYB4-R	ACAACCCTCCATTAATGCCGAC		qRT-PCR
KJ020111	McMYB5-F	GACGCCGTGTTGCGTAAAG	339 bp	qRT-PCR
	McMYB5-R	TCTTCTTCATCGGGGGCGA		qRT-PCR
KJ126859	McMYB6-F	AGACTGCCGGGACGAACCGA	85 bp	qRT-PCR
	McMYB6-R	CCTTGTCGCTTCCTTGCTCCGT		qRT-PCR
FJ817489	McMYB7-F	AGGGTGCAAAAACAGGCGCG	84 bp	qRT-PCR
	McMYB7-R	CGGCACCCAGAAACCCCGAAC		qRT-PCR
KJ020112	McMYB12-F	GATAACAGCGTCGTCCTCGTC	104 bp	qRT-PCR
	McMYB12-R	CGGGAGTCCAAGGCCCTC		qRT-PCR

qPCR analysis was carried out in a total volume of 20 µl containing 9 µl of 2×SYBR Green qPCR Mix (Takara, Japan), 0.1 µM specific primers (each), and 100 ng of template cDNA. The reaction mixtures were heated to 95°C for 30 s, followed by 39 cycles at 95°C for 10 s, 59°C for 15 s, and 72°C for 30 s. A melting curve was generated for each sample at the end of each run to ensure the purity of the amplified products.

### Data analysis

All data were analyzed using one-way ANOVA followed by Duncan's SSR test (shortest significant ranges) to compare differences among the experimental sites at *P*<0.05 (Microsoft Excel 2003 and Data Processing System (DPS) software 7.05).
